# Functionalization of Crosslinked Sodium Alginate/Gelatin Wet-Spun Porous Fibers with Nisin Z for the Inhibition of *Staphylococcus aureus*-Induced Infections

**DOI:** 10.3390/ijms22041930

**Published:** 2021-02-16

**Authors:** Natália C. Homem, Tânia D. Tavares, Catarina S. Miranda, Joana C. Antunes, M. Teresa P. Amorim, Helena P. Felgueiras

**Affiliations:** Centre for Textile Science and Technology (2C2T), Department of Textile Engineering, University of Minho, Campus of Azurém, 4800-058 Guimarães, Portugal; taniatav@2c2t.uminho.pt (T.D.T.); catarina.miranda@2c2t.uminho.pt (C.S.M.); joana.antunes@2c2t.uminho.pt (J.C.A.); mtamorim@det.uminho.pt (M.T.P.A.); helena.felgueiras@2c2t.uminho.pt (H.P.F.)

**Keywords:** antimicrobial peptide, biodegradable microfibers, calcium chloride, glutaraldehyde crosslinking, microfiber functionalization, bactericidal action

## Abstract

Nisin Z, an amphipathic peptide, with a significant antibacterial activity against Gram-positive bacteria and low toxicity in humans, has been studied for food preservation applications. Thus far, very little research has been done to explore its potential in biomedicine. Here, we report the modification of sodium alginate (SA) and gelatin (GN) blended microfibers, produced via the wet-spinning technique, with Nisin Z, with the purpose of eradicating *Staphylococcus aureus*-induced infections. Wet-spun SAGN microfibers were successfully produced at a 70/30% *v*/*v* of SA (2 wt%)/GN (1 wt%) polymer ratio by extrusion within a calcium chloride (CaCl_2_) coagulation bath. Modifications to the biodegradable fibers’ chemical stability and structure were then introduced via crosslinking with CaCl_2_ and glutaraldehyde (SAGNCL). Regardless of the chemical modification employed, all microfibers were labelled as homogeneous both in size (≈246.79 µm) and shape (cylindrical and defect-free). SA-free microfibers, with an increased surface area for peptide immobilization, originated from the action of phosphate buffer saline solution on SAGN fibers, were also produced (GNCL). Their durability in physiological conditions (simulated body fluid) was, however, compromised very early in the experiment (day 1 and 3, with and without Nisin Z, respectively). Only the crosslinked SAGNCL fibers remained intact for the 28 day-testing period. Their thermal resilience in comparison with the unmodified and SA-free fibers was also demonstrated. Nisin Z was functionalized onto the unmodified and chemically altered fibers at an average concentration of 178 µg/mL. Nisin Z did not impact on the fiber’s morphology nor on their chemical/thermal stability. However, the peptide improved the SA fibers (control) structural integrity, guaranteeing its stability for longer, in physiological conditions. Its main effect was detected on the time-kill kinetics of the bacteria *S. aureus*. SAGNCL and GNCL loaded with Nisin Z were capable of progressively eliminating the bacteria, reaching an inhibition superior to 99% after 24 h of culture. The peptide-modified SA and SAGN were not as effective, losing their antimicrobial action after 6 h of incubation. Bacteria elimination was consistent with the release kinetics of Nisin Z from the fibers. In general, data revealed the increased potential and durable effect of Nisin Z (significantly superior to its free, unloaded form) against *S. aureus*-induced infections, while loaded onto prospective biomedical wet-spun scaffolds.

## 1. Introduction 

Microbial resistance against conventional antibiotics is being classified as a public health problem, with enormous economic consequences worldwide [1,2]. According to the Organization for Economic Cooperation and Development (OECD), 2.4 million people in Europe, North America, and Australia are predicted to die from infections caused by resistant microorganisms in the next 30 years, with an associated economic cost of approximately US$3.5 billion per year [3]. *Staphylococcus aureus* induced infections are among the most prevalent. *S. aureus* is a common opportunistic pathogen, which resistance to multiple antibiotics has moved it to the front of the line of the World Health Organization (WHO) concerns [4]. Both the academia and the industry sectors have been devoting their efforts in finding solutions or alternatives to the widespread antibiotic resistance of this bacterium [5,6,7,8,9,10]. In this scenario, antimicrobial peptides (AMPs) are gaining more importance in pharmacology or biomedicine strategies as they can target multiple organisms with efficiency, are less likely to induce resistance, and are not just bacteriostatic but also bactericidal [11,12,13]. 

AMPs can be divided in three major classes: class I, which comprises the lantibiotic peptides (typical size < 5 kDa); class II, which consists of the non-modified, heat-stable peptides (<10 kDa); and class III, which encompasses the heat labile and generally larger in size peptides (c.a. 30 kDa). Among those, Nisin, a class I AMP produced by the non-pathogenic bacteria *Lactococcus lactis*, is known to exhibit great inhibitory effects towards Gram-positive pathogenic bacteria. Nisin, the only bacteriocin classified as “Generally Recognized as Safe” (GRAS) by the Food and Drug Administration (FDA), is a polycyclic peptide composed of 34 amino acids with a size of 3.5 kDa [14,15]. It has two main variants: Nisin A and Nisin Z. The main difference between the two is in the amino acids that occupy the 27th position, namely histidine and asparagine for Nisin A and Nisin Z, respectively. This difference does not affect the peptide antimicrobial activity but rather interferes with its solubility at neutral pH; Nisin Z is more soluble than Nisin A since the asparagine amino acid has a more polar side chain than histidine [16,17]. Further, as the isoelectric point of Nisin Z varies between 8 and 9, which makes the peptide positively charged at neutral pH, interactions with negatively charged natural-origin polymers, such as sodium alginate (SA), can be achieved via electrostatic interactions. These conjugations with polymers may provide Nisin Z with protection against degradation by biological fluids or biocomponents (i.e., enzymes), thus maintaining its antimicrobial potential for an optimal, target-directed action against pathogenic bacteria, including *S. aureus* [18]. 

SA is a natural linear polysaccharide derivative of alginic acid (AAc), obtained from brown seaweeds, and composed of 1,4-linked-β-D-mannuronic (M) and α-l-guluronic (G) monomers [19]. The composition of SA, namely the ratio of the two uronic acids and their sequential arrangements, varies according to the extraction source. It usually contains approximately 30 to 60% of AAc, with the conversion of AAc into SA being responsible for its solubility in water. SA is a non-toxic, biodegradable, and biocompatible polymer, capable of retaining large amounts of water [20,21]. It can be easily processed via wet-spinning technique in the form of continuous, uniform microfibers using coagulation baths containing divalent cations, such as calcium (Ca^2+^), which allow for specific and strong interactions to be formed between the long stretches of the G units and the divalent cations [22,23]. However, SA fibers are mechanically weak in wet conditions. To overcome such problematics, SA has been blended with other biopolymers such as gelatin (GN), which is also biocompatible, edible and water soluble [24]. 

GN is a biodegradable protein obtained via the thermal denaturation or the physical and chemical degradation of collagen. It is very abundant in nature and relatively low cost [25]. In combination with SA, GN’s adhesiveness has been known to increase, turning it desirable for biomolecule or drug binding (carrier) [22]. As both SA and GN are water soluble, to serve as drug delivery platforms and guarantee a sustainable, prolonged release, the polymers are required to undergo a crosslinking process. SA crosslinking occurs spontaneously during wet-spinning process, once that the extrusion is performed into a solution containing divalent cations. On the other hand, GN usually requires the action of crosslinking agents such as glutaraldehyde [26]. 

Several delivery systems have been designed and engineered to attain a controlled, sustained release of Nisin, including Nisin Z, for food industry applications [18,27,28,29]. However, to the authors’ knowledge, no reports have been published on the functionalization of wet-spun microfibers with Nisin Z to serve as platforms in the fight against *S. aureus*-derived infections. In this context, the aim of this study was to evaluate, in a first instance, the compatibility of the polymers, SA and GN, during wet-spinning processing, and then to modify their surface with Nisin Z and map their controlled release kinetics and antimicrobial action. Single- and dual-polymer fibers were produced and examined for their loading capacity and availability of the biomolecule while in contact with the Gram-positive bacteria [30]. The wet-spun fibers were characterized for their physical, chemical, and thermal properties. The long-term stability of the fibers was evaluated in physiological media up to 28 days of incubation [31]. Antimicrobial testing was conducted in dynamic conditions. 

## 2. Materials and Methods

### 2.1. Materials

SA (alginic acid sodium salt from brown algae, medium viscosity) and GN (gelatin, type A from porcine skin, ≈300 bloom) were obtained from Sigma-Aldrich (St. Louis, Missouri, USA). Calcium chloride (CaCl_2_, anhydrous) was employed as coagulation agent and was acquired from Chem-Lab (Zedelgem, Belgium). Sodium phosphate dibasic (Na_2_HPO_4_, Sigma-Aldrich), monosodium phosphate monohydrate (NaH_2_PO_4_.H_2_O, Merck, Darmstadt, Germany) and sodium chloride (NaCl, Merck) were used in the preparation of phosphate buffer saline solution (PBS at 0.25 M: 30 g/L of Na_2_HPO_4_, 5.5 g/L of NaH_2_PO_4_.H_2_O and 212.5 g/L of NaCl). Simulated body fluid (SBF) was prepared following the recipe of Srinivasan et al., with all components being purchased from Sigma-Aldrich and used without further purification [32]. Glutaraldehyde solution (grade II, 25% in water) was acquired from Sigma-Aldrich and used as crosslinking agent. Nisin Z peptide (Mw 3331.05, potency < 38,000 IU/mg) was obtained from Toku-E (Sint-Denijs-Westrem, Belgium). Gram-positive bacteria *S. aureus* (ATCC 6538) was supplied by American Type Culture Collection (ATCC, Manassas, Virginia, USA). For bacteria growth and preservation, trypticase soy broth (TSB: 15 g/L of peptone from casein, 5 g/L of soy peptone and 5 g/L of NaCl) and trypticase soy agar (TSA: 15 g/L of peptone from casein, 5 g/L of soy peptone, 5 g/L of NaCl and 15 g/L of agar) media were purchased from VWR (Alfragide, Portugal). Mueller Hinton broth (MHB: 17.5 g/L of acid casein peptone, 2 g/L of beef infusion and 1.5 g/L of maize starch) was obtained from CondaLab (Madrid, Spain). 

### 2.2. Examinations of Nisin Z Action in Free State

#### 2.2.1. Minimum Inhibitory Concentration (MIC) of Nisin Z

MIC of Nisin Z for *S. aureus* bacteria was assessed by the broth microdilution method [33]. Initially, a stock solution of Nisin Z (concentration 1 mg/mL) was prepared by dissolving the peptide in distilled water (dH_2_O), for two minutes, using an ultrasonic bath. Then, 100 μL of this solution were added to the first column of a 96-well plate (in triplicate). Subsequently, serial dilutions (1:2) were performed in MHB, reaching a final volume of 50 μL in each well. A bacteria suspension prepared in MHB at a concentration of 1 × 10^7^ colony forming units (CFUs)/mL was then added to the wells (50 μL). As control, free bacteria suspensions (positive) and MHB (negative) were used. Absorbance measurements were performed with an EZ READ 2000 Microplate Reader (Biochrom, UK) at a wavelength of 600 nm, at time 0 h and after 24 h of incubation at 37 °C and 120 rpm. The MIC was established as the concentration at which the growing of bacteria was no longer sustained and was detected either visually or through the difference in absorbance values. The minimum bactericidal concentration (MBC) value was determined by culturing the bacteria solution at MIC and its vicinities (before and after MIC value). To accomplish that, aliquots were collected, serially diluted in PBS (10^1^ to 10^4^), plated in TSA, and then incubated at 37 °C for 24 h, at which point grown colonies were observed and counted. 

#### 2.2.2. Agar-Well Diffusion Assay of Polymer Solutions and Nisin Z

The antibacterial activity of SA, GN, SAGN, and Nisin Z solutions was assessed against *S. aureus* via the Kirby–Bauer method [2]. Solutions were prepared in the concentrations employed for fiber production: 2 wt% for SA and ≈1 wt% for GN, and then combined at a polymer ratio of 70:30 *v*/*v* (for SA and GN, respectively). Nisin Z was examined at MIC, 2 × MIC and 3 × MIC. 0.33 mL of the bacterium inoculum, adjusted to 1.0 × 10^7^ CFUs/mL in TSB, were collected and added to 4.67 mL of TSA warmed at approximately 45 °C. The 5 mL bacterial solution was casted into 55 mm diameter sterilized Petri dishes. After agar solidification, 6 mm diameter holes were made at the center of each bacteria-containing agar plate using a sterilized puncher, and 40 μL of each testing solution were introduced in the respective holes. Plates were incubated at 37 °C for 24 h. After this period, zones of inhibition (ZoIs) were observed and measured (mm) to verify the antibacterial efficiency of the solutions. 

### 2.3. Wet-Spun Fibers Production 

SAGN fibers were produced by mixing SA and GN at a polymer ratio of 70:30 *v*/*v*. These solutions were prepared by dissolving SA (2 wt%) and GN (≈1 wt%) separately, in dH_2_O, at 50 °C under stirring for 3 h and 1 h, respectively. After, the GN was added to the SA solution (for the SAGN fibers) and the mixture was left under slow stirring until a homogeneous blend was obtained (1 h). The dissolution step was followed by ultrasonication for 1 h, to remove air bubbles. Once the blends complete dissolution and degassing was achieved, the fibers were produced by wet-spinning using a set-up composed of a syringe pump (NE-1600, New Era Pump Systems, Norleq, Santo Tirso, Portugal), to control the rate and volume of extrusion, and a tray containing the selected coagulation bath. A 20 mL syringe and an 18-gauge (G) needle were coupled to the pump, maintaining a distance between the needle tip and the bottom of the coagulation bath of ≈3 cm. The spinning was performed at a rate of 0.1 mL/min and room temperature (RT), directly into the coagulation bath formed of a 2 wt% CaCl_2_ aqueous solution (T ≈ 21 °C). The fibers were collected manually and stored in a cabinet desiccator (Sicco, Grünsfeld, Germany) at 19 °C and a relative humidity of 41%. Control fibers made exclusively of SA were also extruded using equal processing conditions and stored. Continuous GN fibers could not be extruded directly from a GN-single solution due to their quick precipitation and the consequent brittle nature in contact with the coagulation bath. 

#### 2.3.1. Production of SA-Free Fibers and Crosslinking of SAGN Fibers

SA-free fibers were produced via the removal of SA from SAGN fibers, and subsequent crosslinking with glutaraldehyde (used for GN). Two different approaches were applied: in one the SAGN fibers were immersed in dH_2_O and in another they were submersed in a concentrated PBS solution (0.25 M, pH 7.2). In both cases, fibers were left in contact with the solutions for 48 h at 4 °C. Fibers treated with dH_2_O remained unaltered, while those treated with PBS loss the SA portion of the fiber [30]. Chemical crosslinking was applied to these two groups. For that, fibers were firstly washed in dH_2_O and then immersed in a fresh PBS solution (0.25 M, pH 7.2) containing 2.5 wt% of glutaraldehyde for 1 h. All traces of glutaraldehyde were eliminated by evaporation, followed by successive washings with 0.9 and 0.7% *w*/*v* sodium chloride solutions and dH_2_O. In the end, the crosslinked SAGN fibers were labelled SAGNCL, while those free from SA were labelled GNCL. All samples were dried and stored in a controlled environment (cabinet desiccator) for subsequent testing. 

#### 2.3.2. Nisin Z Functionalization

Fibers were immersed in Nisin Z at an initial concentration of 3 × MIC (pH 6) and left under slow stirring (120 rpm) for 72 h at RT. This concentration was selected to guarantee a proper functionalization of the fibers with the peptide, reaching values of at least 2 × MIC, and was determined based on data collected in previous studies [2,34]. In addition, in these works it was also seen that 2 × MIC or MIC concentrations did not reach similar results to those obtained with the free peptide. Thus, in order to approximate that data, 3 × MIC was required. The pH of the Nisin Z solution (pH 6) was not adjusted so that the peptide maintained its positively charged nature and electrostatic interactions with the polymers would be facilitated [18]. Aliquots of the solution were collected, and their absorbance was measured using an UV–visible spectrometer (UV–VIS 1800, Shimadzu, Switzerland), every 24 h, at 205 nm as stated in the literature [35,36]. This wavelength was also confirmed as the one in which nisin Z presents the maximum absorbance value by scans ranging from 200 to 800 nm. The percentage of Nisin Z incorporated within the fibers was determined by measuring the difference of absorbance at times 0 and 72 h. After this period, the fibers were washed in dH_2_O for 5 min, dried and stored for future testing (Table 1). 

### 2.4. Physical and Chemical Characterization 

#### 2.4.1. Brightfield Microscopy

The morphology of the fibers was verified by brightfield microscopy using an inverted Leica DM IL LED microscope (Leica microsystems, Weetzlar, Germany). Images were taken at 10 × magnification, and the average fiber diameter was determined via ImageJ software (version 1.53) using 5 images per sample (5 measurements per image). 

#### 2.4.2. Attenuated Total Reflectance-Fourier Transform Infrared Spectroscopy (ATR-FTIR)

The surface chemistry and chemical composition of the fibers were analyzed by ATR-FTIR. The equipment used was an IRAffinity-1S (Shimadzu, Kyoto, Japan), coupled with a HATR 10 accessory with a diamond crystal. Spectra were obtained in a wavenumber range of 400–4000 cm^−1^, in a velocity scan of 200 scans at a resolution of 8 cm^−1^.

#### 2.4.3. Thermogravimetric Analysis (TGA)

TGA was conducted to evaluate the variations in the fiber’s thermal stability. Samples were placed in platinum crucibles and measurements were conducted on a STA 7200 Hitachi (Fukuoka, Japan). The temperature ranged from 25 to 500 °C with an increase of 10 °C/min. Measurements were performed under nitrogen atmosphere at 200 mL/min.

#### 2.4.4. Differential Scanning Calorimetry (DSC)

DSC analyses were conducted in a Mettler Toledo equipment model DSC-822 (Columbus, USA). Samples were placed in an aluminum crucible and exposed to a heating gradient of 10 °C/min, at a range of 25 to 500 °C, under nitrogen atmosphere (200 mL/min).

#### 2.4.5. Degradation in Simulated Body Fluid

The fibers degradation profile over time was monitored in SBF at pH 7.4 (physiological conditions) [37]. Samples of 10 mg were incubated in 1 mL of SBF at 37 °C, up to 28 days. Experiments were conducted in static conditions with media being exchange every week. After 1, 3, 7, 14, 21, and 28 days of incubation, the excess of liquid was removed from the samples’ surface using kimwipes (Kimtech) and the samples were weighed. Degradation, measured by mass loss, was calculated using the following equation:

$$\mathrm{mass}\;\mathrm{loss}\;(\%)=\frac{m_{\mathrm{ti}}-m_{\mathrm{tf}}}{m_{\mathrm{ti}}}\times 100,$$

in which *m_ti_* (mg) is the weight of the fiber at day 0 (before sample immersion in SBF) and *m_tf_* is the weight of the fiber after each incubation period. 

### 2.5. Nisin Z Release Kinetics 

The release of Nisin Z from the fibers during time was assessed in physiological conditions (SBF, pH 7.4). Samples of Nisin Z-loaded fibers weighing 10 mg each were left in contact with 1 mL of SBF at 37 °C and 120 rpm, and after 1, 2, 4, 6, and 24 h, aliquots of 50 μL were collected and their absorbance measured via UV–visible spectroscopy. The release of Nisin Z was determined by the differences in absorbance (205 nm) between the first moment of interaction (0 h) and the subsequent measuring periods up to 24 h.

### 2.6. Bacteria Inhibition

To evaluate the efficiency of the Nisin Z-incorporated fibers throughout time, a *S. aureus* suspension, with a concentration of 1 × 10^5^ CFUs/mL in MHB was prepared. Samples of 10 mg were immersed in 1 mL of suspension. Unloaded fibers and free Nisin Z (at 0.5 × MIC, MIC, 2 × MIC and the fiber loading concentration) were used as control. Samples were incubated at 37 °C and 120 rpm, and at pre-determined time points, 1, 2, 4, 6, 24, and 48 h, aliquots were collected. These aliquots were then serially diluted in PBS (10^1^ to 10^4^), plated in TSA, and incubated at 37 °C for 24 h. The grown colonies were counted, and results were expressed in log reduction. All measurements were performed in triplicate and the results were processed using the GraphPad Prism 7.0 software.

## 3. Results and Discussion

### 3.1. Nisin Z MIC against S. aureus

MIC is expressed as the smallest concentration required from an antimicrobial agent to display inhibition against a microbial cell [33]. The MIC value obtained for Nisin Z against the Gram-positive bacterium *S. aureus* was established at 62.5 µg/mL (Table S1 in Supplementary Materials), meaning that this peptide is quite effective against this bacterium. The MIC was determined equal to the MBC, since at this concentration no grown colonies were detected after 24 h of culture (at smaller concentrations those were observed). One of the crucial structures for bacteria survival is its membrane integrity. Gram-positive bacteria, such as *S. aureus*, exhibit a bilayer membrane, the cytoplasmatic membrane, which is surrounded by the cell wall and a thick layer of peptidoglycans (on the outside of the cell membrane) connected to lipoteichoic acids. These components endow the cell with an anionic and amphiphilic character [38]. Nisin Z which is positively charged and possesses amphipathic characteristics displays a unique mechanism of action against this bacterium. It kills by electrostatic binding via a specific membrane target on the *S. aureus*, the Lipid II (amphipathic peptidoglycan that works as a precursor molecule in the synthesis of the cell wall of bacteria, serving as a lipid anchor for many biomolecules). As Nisin Z binds the headgroups of the Lipid II, the membrane domain becomes entrapped by the formation of a surrounding pyrophosphate cage, which affects the cell wall biosynthesis by interrupting the multi-enzymatic peptidoglycan production cycle. Here, the two lanthionine rings of the Nisin Z N-terminus connect with the Lipid II, while the flexible central region of the peptide drives the penetration of its C-terminus portion into the membrane, giving rise to stable pores that ultimately lead to cell lysis. This mechanism of action is known as the pore-forming and classifies Nisin Z as a pore-forming class I bacteriocin agent [17,39].

### 3.2. Agar-Well Diffusion

**Figure 1:**
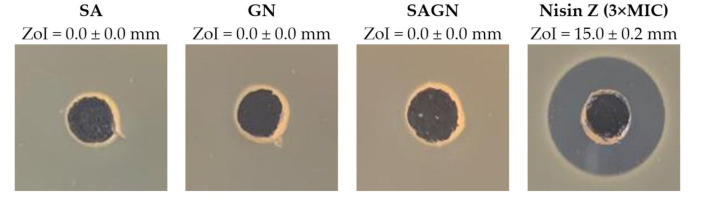
Zones of inhibition (ZoIs) of SA, GN, SAGN and Nisin Z against *S. aureus* (*n* = 3, mean ± standard deviation (SD)). The diameter of the holes (Ø = 6 mm) was included in the measurement of the ZoI formed. Images were collected without regard for size proportionality, only being used as representations of the halos formed.

The antimicrobial activity of the polymeric solutions SA, GN, and their combination SAGN, prepared in dH_2_O at their fiber-producing concentration, was evaluated against *S. aureus* via the agar-well diffusion assay. Nisin Z in its free form was also examined at 62.5 µg/mL (MIC), 125 µg/mL (2 × MIC) and 187.5 µg/mL (3 × MIC) concentrations (Figure 1). The goal was to identify the ability of these solutions to inhibit bacterial action on their own. Data from Table 2 shows the observed ZoIs. As expected, the polymers in their single and dual blend form did not affect the cells viability. ZoIs were only detected in contact with Nisin Z, increasing proportionally with the concentration, from ≈13 to ≈15 mm (Figure S1 in Supplementary Materials). The dimension of the formed halos classifies the Nisin Z antibacterial action as moderated (ZoI between 20 and 12 mm) [40]. Nisin Z at 3 × MIC was selected as representative of this action in Table 2, since this concentration was the one employed during wet-spun fiber functionalization to guarantee a proper and complete binding of the peptide.

### 3.3. Microfibers’ Morphology and Functionalization

**Figure 2:**
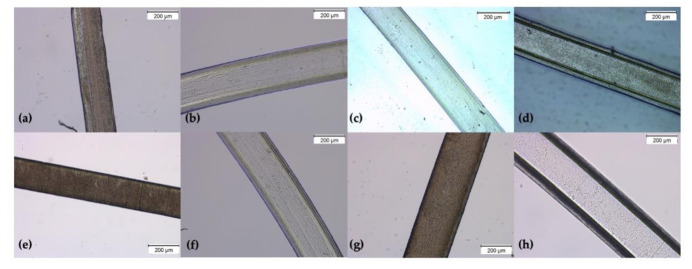
Micrographs of the unloaded and loaded wet-spun fibers (**a**) SA, (**b**) SAGN, (**c**) SAGNCL and (**d**) GNCL, (**e**) SAz, (**f**) SAGNz, (**g**) SAGNCLz, (**h**) GNCLz, captured at 10× magnification using a brightfield microscope. The appearance of a coating-like layer surrounding the fibers is due to the microscope inability to perceive 3D structures, without losing focus.

Microfibers were successfully processed by wet-spinning from polymer solutions of SA and SAGN. Crosslinking was accomplished afterwards to produce SAGNCL and GNCL. Due to its brittle nature, GN solutions could not be wet-spun and used as control. Processing conditions were optimized to obtain continuous and uniform microfibers. Figure 2 shows the resulting morphology of each sample, including after crosslinking. As evidenced, all produced fibers were very homogeneous, displaying a rounded, cylindrical-like shape to the naked eye (Figure S2 in Supplementary Materials). Minor slopes could be observed (Figure 2a); however, considering that for each experiment samples of 10 mg were used, which corresponded to an average fiber length of 125 cm, these small imperfections were neglected. 

The average fiber diameters were determined from 50 measurements conducted in different regions of each type of fiber. Data reported an average diameter of 228.2 ± 5.1 μm for SA, 278.5 ± 3.2 μm for SAGN, 221.0 ± 6.3 μm for SAGNCL and 259.2 ± 4.8 μm for GNCL. The increase in diameters from 228 to 278 μm from the SA to the SAGN samples is easily explained by the conjugation of the two polymers, which allowed the fibers to expand in size by the intermolecular bonds generated. However, after crosslinking, the diameter of the SAGNCL fibers reduced. Because the bonds between the polymers are strengthened, the polymer network becomes tighter and more compact. This also reduces relatively the fibers ability to interact with the air humidity and swell [41]. This phenomenon is, however, observed with more clarity on the GNCL. Indeed, after the removal of the SA portion, the surface area of the fibers increased (larger number of free hydroxyl groups) allowing for more interactions to be formed with water molecules [30]. As a result, the average diameter of these fibers is located in between that of the crosslinked (fewer free binding sites) and the non-crosslinked fibers (more binding sites available). Even though all characterization experiments were conducted with dried samples, stored in a controlled water-free atmosphere prior to handling, their strong affinity towards this element has influenced the observations. For the loaded fibers, fiber diameters were established as 223.5 ± 4.0 μm for SAz, 281.2 ± 3.9 μm for SAGNz, 219.3 ± 7.4 μm for SAGNCLz and 263.4 ± 3.6 μm for GNCLz, thus showing absence of significant alterations compared to the unloaded fibers in terms of average diameter and morphological structure (Figure 2e–h).

Nisin Z was immobilized onto the microfibers by immersion in a 3 × MIC concentrated solution. After the 72 h of incubation the concentration of Nisin Z on each testing fiber was determined. It was seen that SA was capable of loading ≈ 187 μg/mL (3 × MIC), SAGN ≈ 176 μg/mL (2.8 × MIC), SAGNCL ≈ 132 μg/mL (2.1 × MIC), and GNCL ≈ 187 μg/mL (3 × MIC). Data suggests that both SA and GNCL adsorbed completely the peptide in solution. In the first case the electrostatic interactions facilitated this loading, while in second the larger surface area obtained after the removal of SA may have allowed for more molecules to bind. As expected, the SAGNCL fibers, due to their intricated polymer network and restricted binding sites, showed a reduced peptide loading. It is likely that the bonding established with these fibers was more complex than simple electrostatic binding, even requiring alterations in the conformation of the peptide for an increased stability [42]. 

### 3.4. Physical and Chemical Characterization 

#### 3.4.1. ATR-FTIR

**Figure 3:**
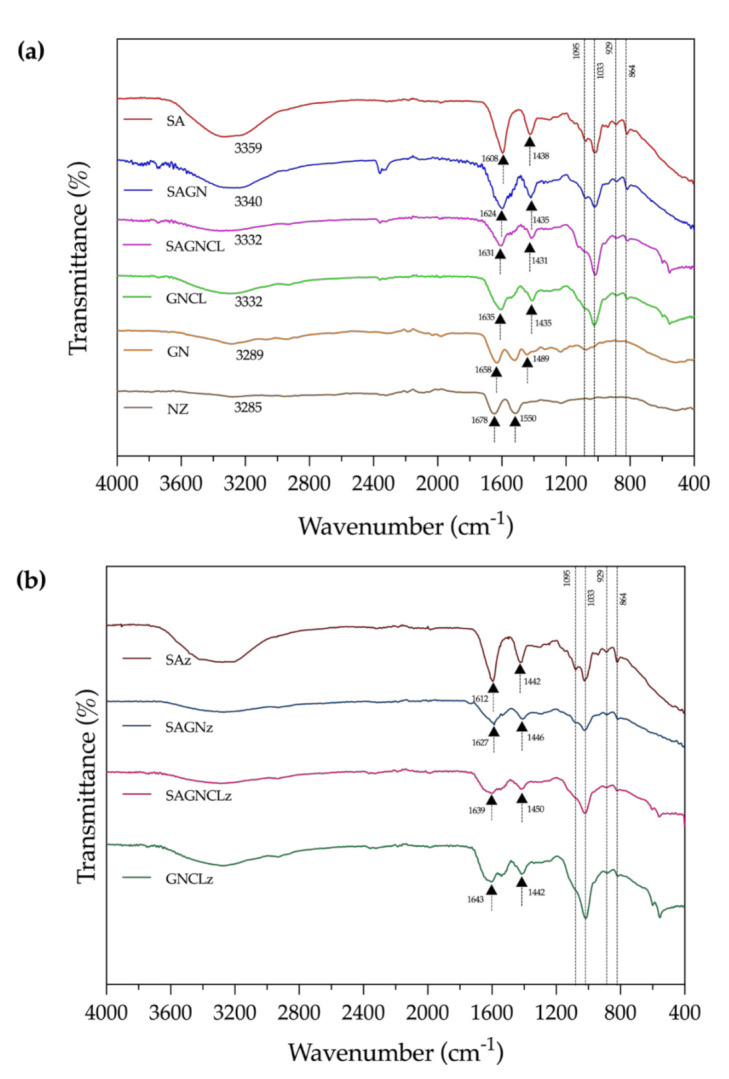
Attenuated Total Reflectance-Fourier Transform Infrared Spectroscopy (ATR-FTIR) spectra of (**a**) SA, SAGN, SAGNCL, GNCL microfibers, GN and Nizin Z powder and (**b**) SAz, SAGNz, SAGNCLz and GNCLz microfibers.

ATR-FTIR spectra of the unloaded and loaded microfibers, as well as GN and Nisin Z powders, were collected (Figure 3a,b). As pointed earlier, control spectra of GN fibers could not be acquired due to their quick precipitation and, consequently, brittle nature in contact with the coagulation bath, which hampered the extrusion process. Modification of the fibers with Nisin Z did not alter the spectra nor introduced any new peaks. Crosslinked fibers did not display any traces of glutaraldehyde, meaning the cleaning process was successful.

A small but wide adsorption peak at approximately 3300 cm^−1^ was observed on all fibers. It referred to the stretching vibrations of intermolecular hydrogen bonds of hydroxyl and/or phenolic groups and may be associated with the exposure of the fibers to the air humidity (an indicative of the affinity of the polymers to water molecules) [2,37]. Due to the crosslinking process, which limited the amount of -OH groups available for binding, the SAGNCL fibers displayed a less pronounced band at this region, attesting to the crosslinking success. For SAGN-containing fibers, regardless of the chemical treatment employed, this band could also be associated with the partially overlapped stretching vibrations of O-H and N-H groups attributed to GN [34]. Characteristic peaks of SA, which presence in the blend was more important, were evidenced between 1608 and 1635 cm^-1^ and between 1431 and 1438 cm^−1^ and were assigned to the asymmetric and symmetric stretching vibrations of the carboxylate salt (COO-) groups. At around 1095 and 1033 cm^−1^, a weak band and a more intense peak were detected and were attributed to the C-O and the C-C stretching vibrations of pyranose ring characteristic of SA [22,43]. Additionally, a band at 929 cm^−1^, assigned to C-O stretching vibration of uronic acid residues, as well as a band at 864 cm^−1^, attributed to C_1_-H deformation vibration of β-mannuronic acid residues, were identified at the anomeric region (1000–750 cm^−1^) [44].

In case of GN powder, as can be observed in Figure 3a, characteristic peaks detected at ≈1658 cm^−1^ and at ≈1489 cm^−1^, can be associated with the amide-I, C-O and C-N stretching vibrations, and with the CH_2_ bend, respectively. The presence of these peaks explains why it was not possible to identify so easily the presence of GN in GN-containing fibers, once that these peaks are overlapped by those of SA (Table S2 in Supplementary Materials). Another peak related to the stretching vibrations of the amines was also observed at ≈1030 cm^−1^ [20,22]. Small shifts in the position of the peaks from the single polymer- to the dual polymer-containing fibers occurred due to the blending and the generation of intermolecular bonds (Figure 3b). For instance, at 1585 and 1573 cm^−1^ new peaks were detected on the dual polymer fibers, particularly on the SAGN and SAGNCL. These peaks confirmed the formation of the polymer complex due to the reaction of the amino groups of GN and the carboxyl groups of SA. Indeed, these peaks could be assigned to the NH_3_^+^ and COO^−^ groups characteristics of the interpolyelectrolyte, which interacted electrostatically forming a stable, highly compatible polymeric blend [20]. Small residues of the presence of SA in the GNCL fibers may have remained after PBS-induced elimination, since very slight protrusions associated with these interactions were detected on this fiber spectrum (e.g., the presence of bands at anomeric region was less intense but still can be identified). This attests to the strength of the intermolecular bonds generated between the two polymers, but also shows that the process employed to eliminate the SA presence from the fibers was not 100% effective [30]. Three of the most important peaks associated with Nisin Z are located at 3288, ≈1640 and 1530 cm^−1^ and are assigned to the O-H asymmetrical stretch, the amide groups and to the bending of the peptide primary amines, respectively. Because of their overlapping with the characteristic peaks of SA and GN and their smaller content when compared to the polymer proportion, no alterations in the ATR-FTIR spectra were noticeable [43].

#### 3.4.2. TGA

**Figure 4:**
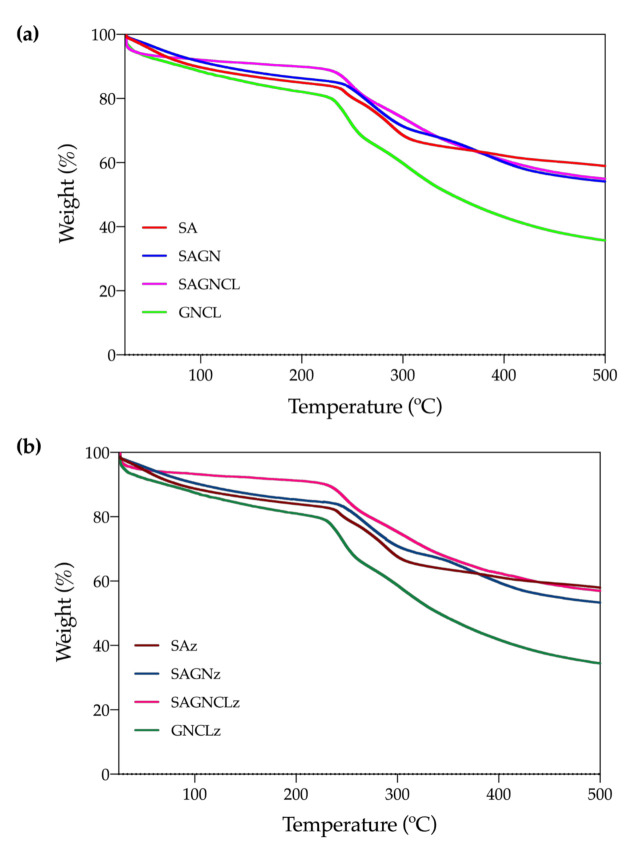
Thermogravimetric Analysis (TGA) curves of (**a**) SA, SAGN, SAGNCL, GNCL and (**b**) SAz, SAGNz, SAGNCLz and GNCLz microfibers, obtained from 25 to 500 °C under nitrogen atmosphere at flow rate of 200 mL/min and temperature rise of 10 °C/min.

Polymer degradation steps associated with temperature rising were identified on the unloaded and loaded microfibers (Figure 4a,b). A first degradation step was recognized on all tested fibers between 75 and 120 °C. It corresponded to the dehydration of the samples (water molecules elimination). Because of the extent of this step, it is likely that both unbounded and bounded water molecules, associated with moisture, to be present on the fibers [45]. As expected from the ATR-FTIR observations (Figure 3), the mass loss experienced by the SAGNCL fibers was the smallest at this point. As both polymers on the blend were crosslinked, generating a more intricated network, fewer hydroxyl groups remained available for water molecules binding. In case of the GNCL, even though crosslinking was also applied, due to the removal of the SA portion of the fiber the surface area augmented and, consequently, the amount of binding sites [30]. Here too, the influence of Nisin Z could not be observed, and as such, very similar degradation curves were obtained for loaded fibers (Figure 4b). Nisin Z is known to exhibit a single degradation step at ≈250 °C. However, it tends to be very feeble even in its powder, highly concentrated free form [43] (due to equipment limitations the peptide powder form could not be analyzed at this time, being this discussion made based on published reports). Around this temperature both polymers SA and GN experience their second degradation peak, losing almost 20% of their mass in all tested fibers. Here, SA decomposes by the dehydration of the saccharide rings, breaking the C-H bonds and the C-O-C glycoside bonds in the main polysaccharide chain, while GN undergoes a complex process in which protein chain breakage and peptide bond rupture occurs [46]. The importance of this decomposition step on the SA and GN polymers explains why the influence of Nisin Z at loading concentration could not be verified. Indeed, considering that the amount of peptide loaded onto the fibers, in terms of mass percentage, is always below 7% of the total polymer mass, it is natural that its detection by TGA or DSC techniques to be unlikely. The third degradation step was identified at around 290 °C for SA and GNCL, at 330 °C for SAGN and at ≈350 °C for SAGNCL. The last one was more difficult to establish since the degradation process of the structural backbone of the SAGNCL fibers progressed at a less pronounced pace from ≈250 °C to 500 °C than the remainder fibers. The presence of crosslinked chemical groups increased the thermal stability of the fibers, turning decomposition more difficult. However, no traces of glutaraldehyde were detected corroborating the ATR-FTIR observations (Figure 3). In fact, aside from eliminating the main chains of the SA and GN, small molecules arising from the crosslinking process, such as CO that worked as an infusible support of the polymers, and the intricated polymeric network generated, needed as well to be eliminated [47]. Regardless, in the end, the percentage of mass that remained was similar to that of the SAGN fibers, ≈60%, and was reduced to carbonaceous materials. As anticipated, the GNCL behaved similarly to pure GN [48]. 

#### 3.4.3. DSC

**Figure 5:**
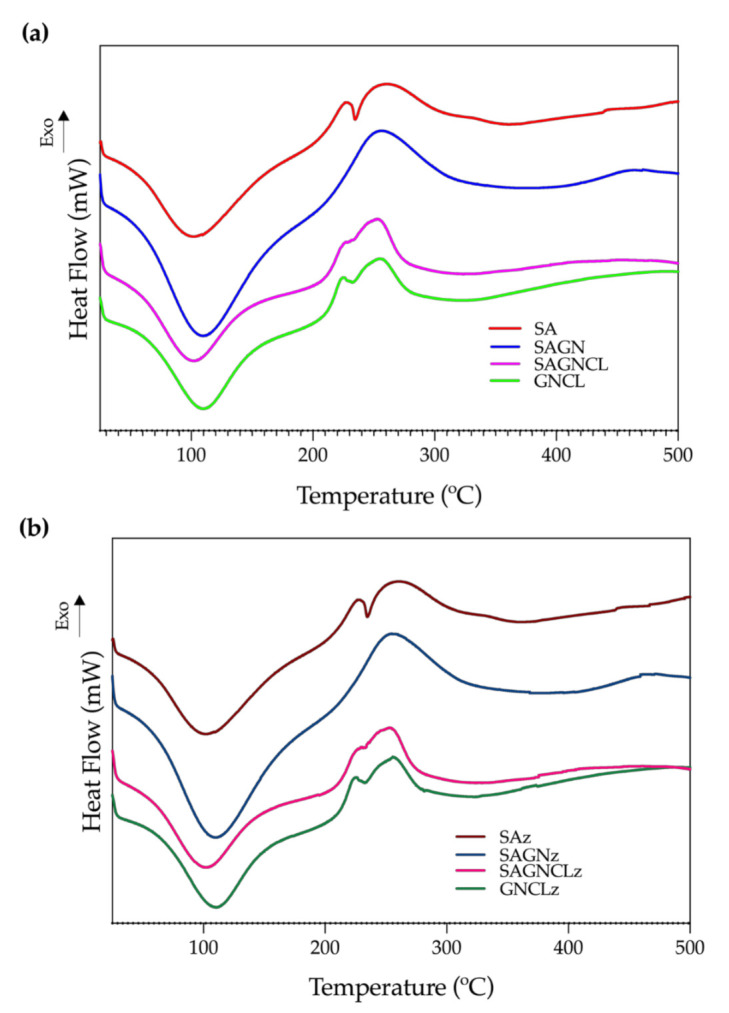
Differential Scanning Calorimetry (DSC) thermograms of the (**a**) SA, SAGN, SAGNCL, GNCL and (**b**) SAz, SAGNz, SAGNCLz and GNCLz microfibers in a temperature range of 25 to 500 °C, performed at a heating rate of 10 °C/min in a nitrogen atmosphere (200 mL/min).

DSC measures the physical and chemical transformations that the polymeric fibers undergo when subjected to heating. Thermograms of the four unloaded fibers were collected and presented in Figure 5. As happened in the TGA analysis, data from the Nisin Z-loaded fibers did not introduce any alterations to the observed curves (Figure 5b). An important endothermic peak was initially detected at ≈110 °C, which was consistent with the first degradation step of TGA. This was correlated with the loss of water molecules linked to the hydrophilic groups of the polymers [45]. A higher exothermic peak was then identified at ≈250 °C and was associated with the degradation, dehydration, and depolymerization reactions conducive to the decarboxylation of the -COOH groups of the SA and the protein chain breakage and peptide bond rupture of GN. At this point, the main skeleton of the polymers starts degrading and subdividing in carbonaceous materials, a phenomena that continues until the 500 °C [43,46]. Indeed, between 300 and 500 °C, the obtained thermograms display another exothermic peak, in some instances barely perceived, that can be attributed to the final degradation of the remaining residual polymeric chains into carbon char [37]. In general, the two main peaks detected were consistent with the observations made with the TGA data. 

#### 3.4.4. Degradation in SBF

The stability of the unloaded and Nisin Z-loaded microfibers in physiological media, namely SBF at pH 7.4, was evaluated for a period of 28 days (Table 2). Considering SBF has osmolarity and ion concentrations that match those of the human body, this medium was selected with the purpose of approximating testing conditions to real-life environments [37]. Results were collected in the form of percentage of mass reduction. As the fibers’ structural integrity became compromised and fragments replaced continuous fibers, weighting was no longer possible due to the significant measuring errors and, as such, pictures were taken. 

Data demonstrated that only the SAGNCL and SAGNCLz fibers could retain their structural integrity throughout the 28 days testing period. They loss between 67 and 61% of their total mass, respectively, but sustained a continuous, elongated fiber morphology. Indeed, no ruptures on the fiber structure were observed. This confirms the successful crosslinking of these samples. Here, the binding of the Ca^2+^ ions from the CaCl_2_ bath with the carboxyl groups from the SA and the aldehyde groups from the glutaraldehyde with the *ε*-amino groups of lysine or hydroxylysine from the GN, have strengthened the intramolecular forces of the polymers, leading them to increase their resistance to prolonged physiological media exposure [41]. However, this was not evidenced on the Nisin Z unloaded and loaded GNCL. In fact, these fibers were extremely weak, losing their integrity from day 1. The presence of the peptide was even considered a compromising factor since the fibers’ resilience decreased. It is likely that the binding sites of the peptide together with the alterations in its conformation and the repulsion between the positive charges of Nisin Z and GN may have led to the appearance of stress sites along the fiber, more easily breakable. Although visually the fibers appeared to be present and to preserve their extended, well defined round-like morphology, upon a close observation it was clear that the tangled fibers were made of fragments and not a continuous single structure. According to Yang et al., the elimination of the SA portion from the fibers leads to the development of an interconnected porous structure. Even though the peptide binding area may increase, the fibers mechanical properties become very feeble [30]. 

The integrity of the SA and SA fibers loaded with nisin, here labeled as SAz, was guaranteed until the 14th day of incubation, while that of the SAGN and SAGNz fibers remained intact only until the 7th day. In case of the first group, the electrostatic interactions between Nisin Z and SA may have preserved the morphology of the fibers for longer. Without the peptide, the fibers became highly hydrated, lost their structural form, and aggregated. Fiber fragments were seen to bind with each other forming heterogeneous, hydrogel-like structures. Similar observations were made with the SAGN fibers. Regardless of peptide presence or absence, continuous fibers became divided in fragments after day 7. This was most important in the absence of Nisin Z since some of those fragments swelled and bonded, forming heterogenous structures. To the contrary, the morphology of the SAGNz remained. Here, swollen fiber fragments were detected. Once again, it is likely that by binding with Nisin Z the structural integrity of the fibers remained for longer. It has been reported that the swelling capacity of SAGN composites is intimately related with the duration of the crosslinking [41]. In this group of samples, intramolecular bonding was achieved in a few seconds of contact with the CaCl_2_ coagulation bath and only by the SA portion. Therefore, it was expected GN to be quickly lost in contact with water-based solutions and SA to follow at a slower pace. The swelling capacity of the peptide unloaded and loaded SA and SAGN fibers may also be behind the boost in mass registered overtime. Indeed, increased swelling at pH 7.4 of SA-based polymer blends has been attributed to the repulsions induced by the ionization of its carboxylate groups [49]. This leads to the expansion of the polymeric network and, consequently, to the fibers increased capacity to swell. Further, this can also lead to new electrostatic interactions with the salt components from the SBF, which would explain the disproportional variations in mass throughout the experiment as water molecules are replaced by salts and vice-versa. 

### 3.5. Nisin Z Release Kinetics

**Figure 6:**
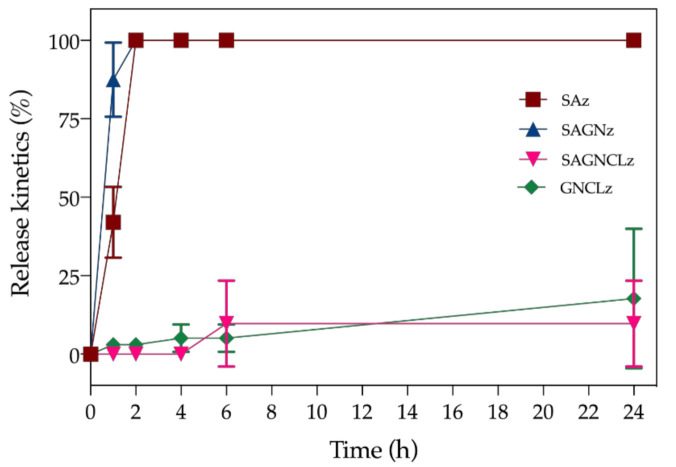
Release kinetics of Nisin Z from SAz, SAGNz, SAGNCLz and GNCLz incubated in SBF (pH 7.4) for a period up to 24 h in dynamic conditions (120 rpm).

The Nisin Z release trend from the SAz, SAGNz, SAGNCLz and the GNCLz functionalized fibers during the 24 h testing period is shown in Figure 6. The rate of peptide diffusion was assessed in SBF and under dynamic conditions, mimicking the physiological environment. The equilibrium of diffusion was attained at 2 h for SAz and SAGNz, and was reached at 6 h by SAGNCLz and at 24 h by GNCLz. It is interesting to notice that, even though the content of Nisin Z immobilized is similar between fibers, the crosslinked ones induced a more controlled and sustained release over time. Indeed, after 24 h of incubation the percentage of peptide release reached only ≈9.7% for SAGNCLz and ≈17.7% for GNCLz. This occurs due to the type of interactions that are formed with the fibers’ surface. In the absence of crosslinking, the fibers display a larger number of free hydroxyl groups, and therefore more physical binding, by means of hydrogen bonding, is allowed. Electrostatic interactions are as well very likely to occur due to the peptide positive charges [18]. Further, the fibers equilibrium of swelling degree is also augmented, thus entrapping more Nisin Z molecules within their structure. In this case, the interactions formed are weaker and more liable to leaching; hence, peptide burst release occurs at the first instances of interaction with the dynamic environment. This is evidenced in the SAz and SAGNz release curves. In the SAGNz, it is also possible this initial burst release to be instigated by the electrostatic repulsion between the positively charged Nisin Z molecules and the positively charged side chains of GN [50]; therefore, explaining the even quicker release of the peptide from the SAGNz (≈87.5%) relatively to the SAz (≈42.0%) after 1 h of incubation. After 2 h, the totality of the peptide is already detached from the fibers. On the other hand, on crosslinked fibers due to the higher network of intra- and intermolecular interactions generated within and between polymers, the hydration capacity of the fibers is reduced, and fewer quick binding sites are made available. Moreover, its inherent solubility is also reduced (Table 2), minimizing the probability of initial burst releases. Here, the crosslinking process strengthened the intramolecular forces of the polymers limiting their organizational freedom for peptide binding [41]. Hydrogen and electrostatic bonding, even though possible, became constraint due to the fewer binding groups available. As such, Nisin Z is left to interact with the surface of these crosslinked fibers via the still accessible carboxyl or amine groups, establishing stronger interactions, less conducive to a quick leaching [51]. Still, it should be noticed that release of the peptide from the GNCLz occurs more quickly than on the SAGNCLz. This can be explained by the larger surface area presented by the SA-free fibers, which also allows more peptide molecules to bind via physical binding, as evidenced for the fiber’s larger diameters (Figure 2) or hydroxyl-based chemical bonding detected on ATR-FTIR (Figure 3). 

### 3.6. Bacteria Inhibition 

**Figure 7:**
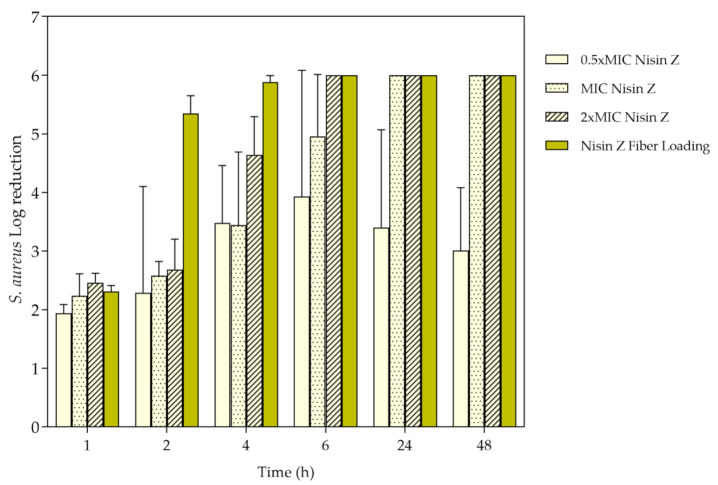
*S. aureus* log reduction in contact with Nisin Z in its free state at 0.5, 1 and 2 × Minimum Inhibitory Concentration (MIC) and at the loading concentration in the fibers, after 1, 2, 4, 6, 24, and 48 h of culture. Data derived from three repetitions (mean ± S.D.).

*S. aureus* growth inhibition was evaluated by the time-kill kinetics for each unloaded and loaded fiber and determined by the number of remaining viable colonies at specific incubation periods, namely 1, 2, 4, 6, 24, and 48 h. To confirm the efficiency of Nisin Z against this microorganism, experiments were also conducted with the free peptide (Figure 7) at 0.5 × MIC, MIC, 2 × MIC and the average fiber-loading concentration (≈178 μg/mL). Data reported a complete elimination (100%) of the bacterium at 24 h for MIC, and at 6 h for 2 × MIC and loading amount. Even though, from the first moments of incubation, the free peptide demonstrated a significant impact against the bacteria which prevailed for the 48 h testing period, at the highest concentrations of free Nisin Z, the 0.5 × MIC was not as successful, being uncapable of eliminating completely the bacteria, as expected (Section 3.1, determination of MBC). 

**Figure 8:**
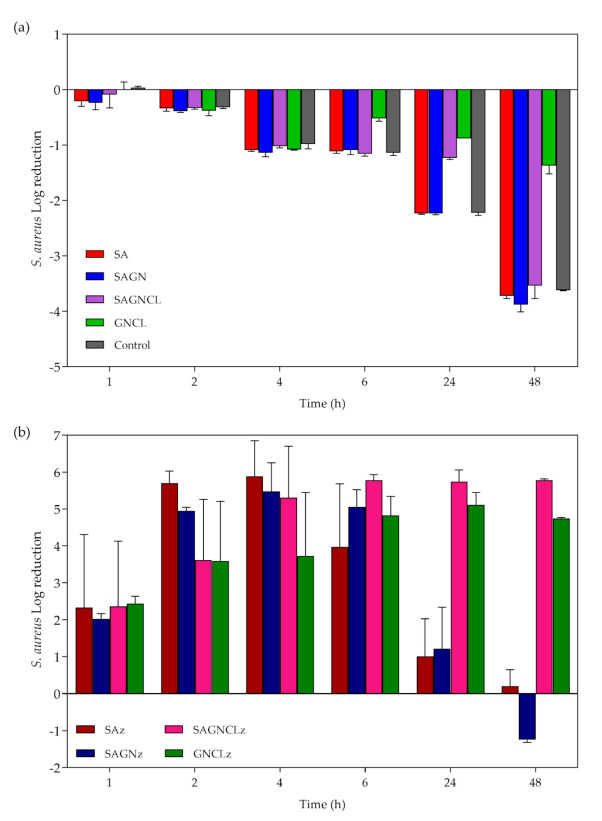
*S. aureus* log reduction in contact with (**a**) unloaded and (**b**) Nisin Z-loaded fibers after 1, 2, 4, 6, 24 and 48 h of culture. Data derived from three repetitions (mean ± S.D.).

Unloaded fibers did not reveal significant inhibitory effects against *S. aureus* (Figure 8a). Indeed, regardless of their composition, the unloaded fibers were incapable of fighting the continuous growth of the bacteria in MHB, throughout the experiment. Between 24 h and 48 h of culture, it was even possible to detect a small improvement in the number of viable cells above the control group (bacteria free from the antimicrobial agent), introduced by the SA-containing fibers. As we saw from the data in Table 2, degradation byproducts are generated from the fibers overtime. Here, it is likely that these residues (e.g., alginic acid and salts) are offering bacteria another source of sustenance, other than the MHB, allowing them to replicate even more [52]. Regarding the loaded fibers, a similar initial performance to that of the free peptide (regardless of concentration) was evidenced for all loaded fibers, with a decrease of ≈99.9% in the total number of viable colonies (Figure 8 b). However, after 24 h of incubation with the bacterial solution, the peptide activity associated with the SAz and SAGNz fibers was lost. Considering the release kinetics of the peptide from these two samples (Figure 6), this lack of bacterial inhibition at this time would be expected. In the first 6 h, *S. aureus* growth inhibition was guaranteed, although never reaching 100% of elimination. Due to the strong influence of the ionic strength of the salt components present in the culturing media, availability of Nisin Z can become limited overtime. Indeed, electrostatic interactions with the salts are likely to occur for the stabilization of the Nisin Z molecule in solution [29,53]. Thus, by not eliminating the bacteria completely and by diminishing its binding sites with interactions with media components, the released Nisin Z was uncapable of stopping bacteria replication, with the remaining microbial cells being able to feed from the degradation byproducts of the fibers and the nutrients in MHB [54,55] and, thus, restore their original levels. These results also demonstrated that a burst release of large amounts of Nisin Z do not guarantee a complete elimination of the microbial cells overtime. Considering the amount of Nisin Z loaded onto the fibers was superior to MIC value, one would expect that after 6 h of incubation all bacteria would be eliminated (Figure 7), regardless of the fibers they were loaded into. However, this did not happen. It is likely that competition between peptide molecules against the binding sites in the bacteria to take place decreasing the killing time rate, and that the release profile of Nisin Z from the fibers may not be entirely reliable since it did not take into consideration the velocity of the replication cycle of the bacterial cells. 

Regarding the fibers that underwent crosslinking, the SAGNCLz and GNCLz, the continuous liberation of the peptide in a sustained way allowed for a more assertive action against this bacterium. During the 48 h, the activity of the GNCLz followed the progression of the release kinetics (Figure 6), culminating in the elimination of ≈99.99% of the microorganism’s presence. Even though release of Nisin Z from the SAGNCLz only started after the 4 h mark, antimicrobial action induced by these fibers was significant from the first hour. Considering the mechanism of action of Nisin Z against *S. aureus* is driven by electrostatic interactions, it is likely that peptide release started earlier in the bacterial suspension than on the SBF (release kinetics studies) [17,39,55]. Once again, we realize that the release profile observed in Figure 6, is a mere indication of what the fibers may be experiencing in culture conditions and cannot be taken for granted. Knowing the positively charged peptide is attracted towards the negatively charged microbial cells and that its solubility increases in neutral pH, this may explain the bacterial elimination detected at 1, 2, and 4 h of incubation. Most importantly, these levels of bacteria inhibition were kept constant along the entire experiment, proving that there is indeed a continuous liberation of peptide molecules and that they remain effective. It is also likely that Nisin Z-loaded fibers to be capable of acting against the bacteria via both diffusion and direct contact. From the observations, SAGNCLz fibers were the most effective in fighting bacteria, while maintaining their integrity. 

One of the main conclusions to drawn from this work, is the ability to sustain an effective release of the peptide and consequent antimicrobial action throughout time, using a wet-spinning drug delivery vehicle. Most importantly, it was verified that a controlled liberation of Nisin Z is more effective in fighting bacteria growth, which can be ensured via fiber loading, than an initial burst release of increased concentration. Even though Nisin Z in its free form reclaimed an 100% inhibition rate against *S. aureus* from the very first moments of interaction (exception 0.5 × MIC), this may not be an entirely successful outcome due to the increased concentration necessary to reach this deed, the instability of Nisin Z in physiological media after prolonged exposure, or the fact that its localized action cannot be defined or controlled, potentially raising the risk of occurrence of side effects when explored in in vivo environments. Indeed, a controlled liberation of the peptide overtime offers a more important outcome over the 48 h period, if considering a dynamic, ever changing environment such in real-life conditions. In in vivo environments, it is not possible to control the localized distribution of an unloaded molecule that can quickly enter the blood stream and affect healthy cells and organ systems. However, by using a delivery platform that can release smaller amounts of the antimicrobial agent, overtime, guaranteeing that only a percentage of loaded peptide is made available, this can guarantee a prolonged action that may be more important and effective in the long run. 

## 4. Conclusions

The affinity and antibacterial action of Nisin Z onto SA-based wet-spun fibers was investigated in this work. Data reported the successful production of SA and SAGN fibers and their chemical modification via crosslinking. Production of SA-free fibers was also accomplished. All testing microfibers were determined homogeneous and continuous, and presented a cylindrical-like shape, which was not influenced by the addition of Nisin Z. The peptide was functionalized during a 72 h period, registering an average loading concentration of 178 µg/mL. The structural integrity of most fibers became compromised during the 28-day period of incubation in SBF. The only exception was SAGNCL that due to its intricated network of intra- and intermolecular bonding, strengthened the chemical structure of the fiber and turned it more compact and resilient (both chemically and thermally). Presence of Nisin Z impacted most significantly on the SA fibers (control), preventing their earlier disintegration in physiological conditions. Nisin Z had its most impactful effect against the bacteria *S. aureus*, particularly when loaded onto the crosslinked samples. It was capable of progressively eliminating the viable cells, reaching an inhibition superior to 99% after 48 h of culture. SAz and SAGNz were not as effective losing their antimicrobial action after 6 h of incubation. The progression in *S. aureus* elimination was consistent with the release kinetics of Nisin Z from the fibers. Overall, data revealed the potential of Nisin Z in fighting *S. aureus*-induced infections, while loaded onto biodegradable crosslinked polymeric scaffolds. The next step in this investigation will be to optimize the controlled release of the peptide for longer periods and from more complex polymer-based biomedical systems.

## Figures and Tables

**Table 1:** Produced fibers and respective treatments employed.

Fiber	SA Removal	Crosslinking	Peptide Immobilization
SA	No	No	No
SAz	No	No	Yes
SAGN	No	No	No
SAGNz	No	No	Yes
SAGNCL	No	Yes	No
SAGNCLz	No	Yes	Yes
GNCL	Yes	Yes	No
GNCLz	Yes	Yes	Yes

Abbreviations: SA—sodium alginate; GN—gelatin; Z—Nisin Z peptide; CL—crosslinking.

**Table 2:** Microfibers degradation profile over a period of 28 days in simulated body fluid (SBF) at pH 7.4. Data is presented in percentage of mass reduction ± S.D. (*n* = 3).

Mass Reduction (%)						
Fibers	Day 1	Day 3	Day 7	Day 14	Day 21	Day 28
**SA**	−23.7 ± 23.7	−9.1 ± 21.5	−14.4 ± 28.1	−2.6 ± 35.3	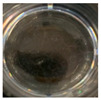	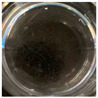
**SAz**	−226.3 ± 64.2	−189.3 ± 42.1	−207.8 ± 40.0	−190.2 ± 39.6	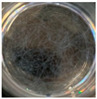	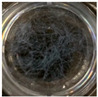
**SAGN**	−115.0 ± 39.2	−106.7 ± 42.7	−112.5 ± 36.5	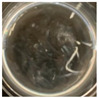	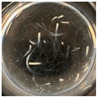	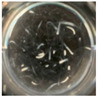
**SAGNz**	−124.0 ± 74.8	−103.8 ± 64.1	−100.4 ± 67.3	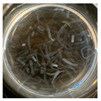	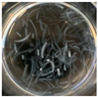	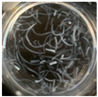
**SAGNCL**	33.9 ± 11.5	45.0 ± 6.8	48.1 ± 1.7	52.4 ± 3.4	55.9 ± 5.8	67.3 ± 14.6
**SAGNCLz**	31.6 ± 5.8	45.3 ± 4.9	49.1 ± 5.2	53.3 ± 5.6	59.7 ± 6.8	61.7 ± 7.8
**GNCL**	53.5 ± 0.5	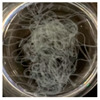	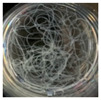	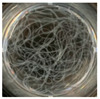	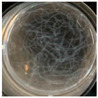	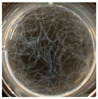
**GNCLz**	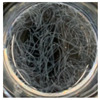	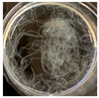	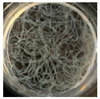	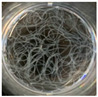	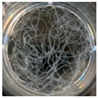	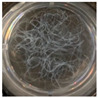

## Data Availability

Not applicable.

## Supplementary Materials

The following are available online at https://www.mdpi.com/1422-0067/22/4/1930/s1.
