# Supplementary material for: Functionalization of Crosslinked Sodium Alginate/Gelatin Wet-Spun Porous Fibers with Nisin Z for the Inhibition of Staphylococcus aureus-Induced Infections
Source: Int J Mol Sci. 2021 Feb 16;22(4):1930. doi: 10.3390/ijms22041930 (PMC7919837; doi:10.3390/ijms22041930)
Supplement: Supplementary file 1 [file ijms-22-01930-s001.pdf]

Supporting Information

Table S1. Absorbance values for MIC determination of Nisin Z.

	Absorbance (600 nm)									
**Concentration (μg/mL)**	**1000**	**500**	**250**	**125**	**62.5**	**31.25**	**15.625**	**7.812**	**3.906**	**1.953**
**0 hours**	0.677	0.718	0.615	0.185	**0.082**	0.071	0.074	0.072	0.081	0.074
**24 hours**	0.209	0.250	0.116	0.092	**0.080**	0.147	0.455	0.393	0.362	0.378

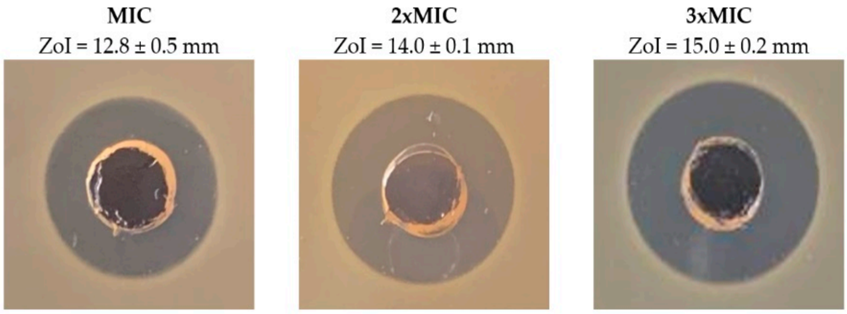

Figure S1. ZoI of Nisin Z solutions at a concentration of (a) 62.5 μg/mL (MIC), (b) 125 μg/mL (2xMIC) and (c) 187.5 μg/mL (3xMIC).

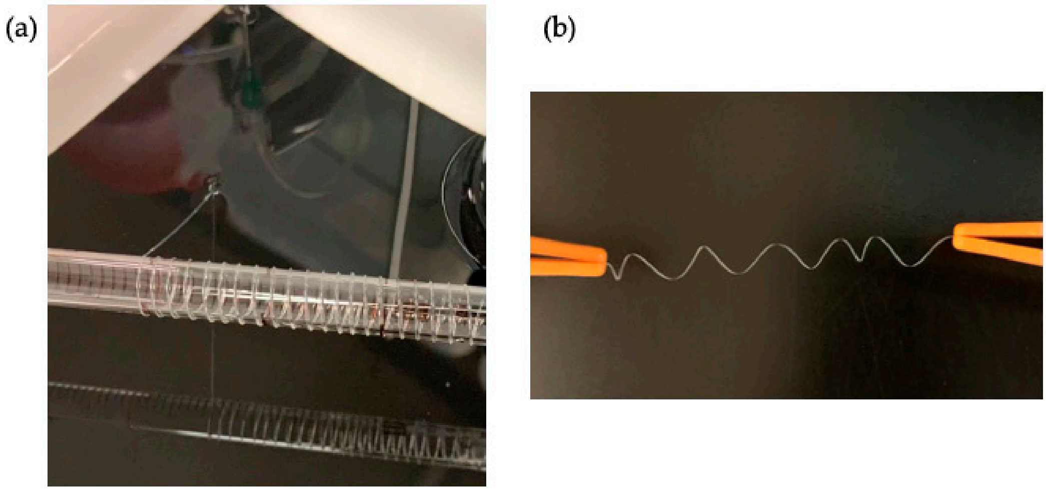

Figure S2. Aspect of the SAGN fibers (a) during and (b) after wet-spinning processing.

Table S2. ATR-FTIR peaks detected and identification of the contribution of a specific component (polymer).

Wavenumber (cm^{-1})	Associated compound, element or sample	Functional group assigned
≈ 3300	All	-OH stretching
1658	GN	amide-I, C-O and C-N stretching vibrations
1640	NZ	Amide groups
1585	SAGN	NH_3^+
1573	SAGN	COO^-
1530	NZ	Primary amines
1489	GN	$-CH_2$ bending
1095	SA	C-O stretching vibration
1033	SA	C-C stretching vibration
1030	GN	Amine groups stretching vibration
1020	SA	O-C-O stretching vibration
929	SA	C-O stretching vibration (uronic acid residues)
864	SA	C-O-C stretching vibration (β-mannuronic acid residues)
1431 - 1438	SA	COO- symmetric stretching
1608 - 1635	SA	COO- asymmetric stretching
